# Comparative evaluation of single camera photogrammetry and LIDAR-based facial scanning

**DOI:** 10.1590/2177-6709.31.4.e2625120.oar

**Published:** 2026-10-12

**Authors:** Aditi SINGH, Payal SHARMA, Piush KUMAR

**Affiliations:** 1 ITS-CDSR, Department of Orthodontics and Dentofacial Orthopaedics (Ghaziabad, Uttar Pradesh, India).

**Keywords:** 3D facial scanning, LiDAR technology, Single-Camera Photogrammetry (SCP), Orthodontic soft tissue analysis, Accuracy and reliability, Escaneamento facial 3D, Tecnologia LiDAR, Fotogrametria de câmera única, Análise de tecidos moles em Ortodontia, Precisão e confiabilidade

## Abstract

**Introduction:**

Orthodontic diagnosis and treatment planning relies on precise diagnostic records, including facial imaging. While two-dimensional (2D) photography is widely used, it cannot fully represent three-dimensional (3D) facial structures. Recent advances in 3D imaging, including LiDAR-based scanning and single-camera photogrammetry (SCP), offer improved accuracy.

**Objectives:**

This study evaluates these cost-effective 3D scanning methods in facial soft tissue assessment, by comparing measurements from SCP and LiDAR-based mobile apps, with manual method measurements as gold standard.

**Methods:**

Thirty-six patients (21 females, 15 males) aged 15-25 years requiring orthodontic treatment participated. Scanning was conducted using SCP and LiDAR technology, while manual measurements used digital calipers. Statistical analysis evaluated accuracy, precision, and reliability.

**Results:**

Both SCP and LiDAR-based scanning techniques show clinically acceptable accuracy in facial soft tissue analysis. LiDAR-based scanning demonstrates better depth perception than SCP, though both methods are viable alternatives to conventional imaging.

**Conclusion:**

The study highlights smartphone-based 3D facial scanning as viable alternatives to traditional imaging in Orthodontics. While SCP and LiDAR scanning show comparable accuracy, further research is needed. The findings support implementing affordable 3D scanning technologies in orthodontic diagnostic protocols.

## RESUMO

**Introdução:**

O diagnóstico e o planejamento do tratamento ortodôntico dependem de registros diagnósticos precisos, incluindo imagens faciais. Embora a fotografia bidimensional (2D) seja amplamente utilizada, ela não consegue representar completamente as estruturas faciais tridimensionais (3D). Os avanços recentes em imagens 3D, incluindo o escaneamento facial baseado em LIDAR e a fotogrametria com câmera única (SCP), oferecem maior precisão.

**Objetivos:**

Este estudo analisou esses métodos de escaneamento 3D de baixo custo para avaliação dos tecidos moles da face, comparando medições obtidas por meio de SCP e aplicativo de celular baseado em LiDAR, tendo como padrão-ouro as medições realizadas manualmente.

**Métodos:**

Trinta e seis pacientes (21 mulheres e 15 homens) com idades entre 15 e 25 anos, que necessitavam de tratamento ortodôntico, participaram do estudo. Os escaneamentos foram realizados utilizando as tecnologias SCP e LiDAR, enquanto as medições manuais foram feitas com paquímetro digital. A análise estatística avaliou a acurácia, a precisão e a confiabilidade.

**Resultados:**

Tanto a técnica de escaneamento SCP quanto a baseada em LiDAR apresentam precisão clinicamente aceitável na análise dos tecidos moles da face. O escaneamento baseado em LiDAR demonstrou melhor percepção de profundidade do que a SCP, embora ambos os métodos sejam alternativas viáveis às imagens convencionais.

**Conclusão:**

O estudo destaca o escaneamento facial 3D usando *smartphones* como uma alternativa viável aos métodos tradicionais de imagens em Ortodontia. Embora os métodos de escaneamento por SCP e LiDAR apresentem precisão comparável, mais pesquisas são necessárias. Os resultados apoiam a implementação de tecnologias de escaneamento 3D acessíveis nos protocolos de diagnóstico ortodôntico.

## INTRODUCTION

Diagnostic records including facial photographs are indispensable for orthodontic diagnosis and treatment planning. It is important to recognize that traditional photographs offer two-dimensional representations of three-dimensional structures. Although, these images cannot replace a meticulous and comprehensive clinical examination of the face. Three-dimensional facial scans, however, can accurately depict facial structures in the absence of the patient and facilitate orthodontic diagnosis and treatment planning.

A variety of techniques are available for generating three-dimensional facial models, including 3D laser scanning, photogrammetry, stereophotogrammetry, structured light, and dual-structured light utilizing infrared sensors.^1^ ^,^ ^2^ Three-dimensional (3-D) stereophotogrammetry facilitates the acquisition of 3-D images by integrating photographs taken from multiple angles using synchronized digital cameras. This technique offers a significant advantage by capturing repeated high-resolution images at short intervals, while minimizing artifacts. Some commercially available options include Di3D (Direct Dimensions) and 3dMD. With ongoing technological advancements, particularly in portability, companies have developed face scanners and software applications (apps) that can be employed on smartphones or tablets to capture 3D patient images in various environments. Single-camera photogrammetry (SCP) systems are cost-effective and require less physical equipment. The app collects data using a small, portable camera that either rotates around the patient’s face or captures images as the patient moves their head in front of a stationary camera. The collected data can then be exported to various software programs or platforms to integrate and fabricate the facial 3D model. Conversely, some smartphones use advanced technology, such as Apple’s LiDAR (Light Detection and Ranging). This technology operates by emitting thousands of tiny, invisible dots of light. When these dots strike the face, they reflect back to the phone, which then analyzes this information to create a detailed map of the face’s shape and depth. This process enables the smartphone to capture 3D images, making it an attractive option for clinicians, particularly in settings where expensive equipment is unavailable. D’Ettorre et al.^3^ stated that the 3dMD device offers rapid and precise face image acquisition but is costly and unwieldy, whereas smartphone applications with TrueDepth sensors provide cost-effective, portable alternatives that require greater operator skill and patient compliance. Staller et al.^4^ compared single and multiple camera 3-dimensional photogrammetry and found that both SCP and MCP (multi-camera photogrammetry) techniques were reliable and valid options for 3D facial imaging. Pellitteri et al.^5^ compared the accuracy of two mobile 3D facial scanning applications, the Dental Pro and the Facial Hunter. They inferred that 3D facial surface scanning has emerged as a reliable method for clinical assessment, offering high accuracy and reproducibility across various scanning technologies. After comparing three specific apps (EM3D, Polycam, and ScandyPro, based on LiDAR technology), Seifert et al.^6^ concluded that smartphone-based facial scan systems could be a viable alternative, particularly in resource-limited settings. However, none of these previous studies directly compared the SCP and LiDAR facial scanning applications. Therefore, the primary aim of this study was to assess and compare the accuracy of low-cost 3D face scanning methods, specifically single-camera photogrammetry and the LiDAR-based 3D facial scanning application available on the iPhone, for the analysis of facial soft tissues.

## MATERIAL AND METHODS

This study was approved by the Institutional Research Ethics Committee. The sample size was calculated from the data obtained from a previous study conducted by D’Ettorre et al^3^, the total sample size estimated was 36 per group at 80% power. Hence, the study population comprised 36 patients (21 females and 15 males), with a mean age of 20±5.3years. Patients who reported to the department were included if they met the following criteria: required fixed orthodontic treatment, aged between 15 and 25 years, and had provided consent to participate in the study. Patients were excluded if they had facial defects, evident facial asymmetry, muscular disorders, a history of facial surgery or trauma, beards and mustaches (for men), scars, or a history of facial aesthetic surgery.

Before capturing facial images using the two distinct 3D imaging systems, black liquid eyeliner was applied directly to each participant’s face, to mark nine standard anthropometric surface landmarks (Table 1, Fig. 1). A single operator provided standardized instructions before each imaging session. During the facial scanning procedures, the participants were seated upright on a chair with back support, maintaining a closed-mouth intercuspal position with relaxed lips. To ensure a natural head position, the participants were instructed to adopt a self-balanced posture by positioning their heads in the most comfortable manner and focusing on a distant point on the wall.

**Table 1:** Definitions of the soft tissue landmarks.

	Name	Abbreviation	Definition
1.	Nasion	N	Deepest point of the nasal bridge
2.	Subnasale	Sn	The point at which the columella (nasal septum) merges with the upper lip in the midsagittal plane
3.	Tragion	t	The point located at the upper margin of each tragus
4.	Pronasale	Prn	Most protruded point of the apex nasi
5.	Cheilion	Ch	Most lateral point of the labial commissure
6.	Pogonion	Pog	Most prominent point of the chin on the sagittal midline
7.	Menton	Me	Most inferior point of the chin on the sagittal midline
8.	Gonion	Go	Most lateral point of the gonial angle contour
9.	Zygion	Zy	The most lateral point on the zygomatic bone

**Figure 1:**
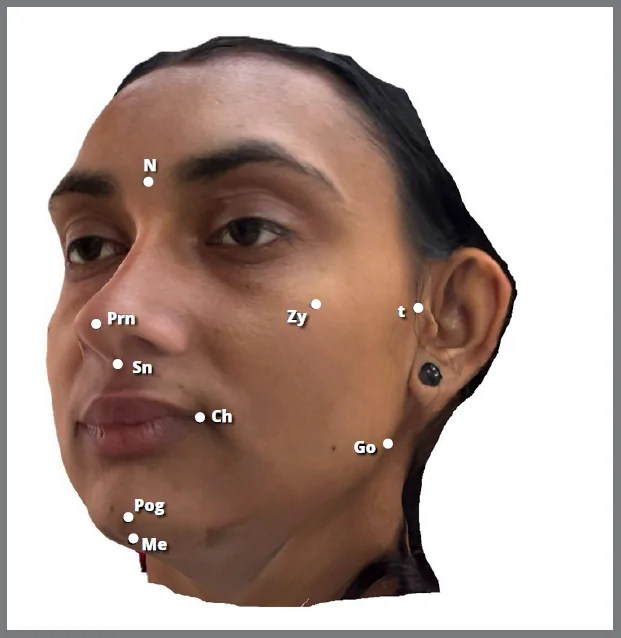
Soft tissue landmarks: N = Nasion; Prn = Pronasale; Sn = Subnasale; Ch = Cheilion; Pog = Pogonion; Me = Menton; Zy = Zygion; t = Tragion; Go = Gonion.

To standardize the lighting conditions, natural light was excluded from the room, and LED front lighting was utilized to ensure optimal facial illumination and minimize shadow formation. All the facial scans were performed on a single day by the same person. Nine soft-tissue landmarks were used to derive 12 linear measurements (Table 2).

**Table 2:** Linear measurements (mm) performed.

1	Nasion-subnasale (N-Sn)	Distance between the soft tissue nasion and subnasale
2	Nasion-pronasale (N-Prn)	Distance between the soft tissue nasion and pronasale
3	Pronasale-menton (Prn-Me)	Distance between pronasale and menton
4	Subnasale-menton (Sn-Me)	Distance between subnasale and menton
5	Interpupillary distance (ITP)	Distance between the pupils
6	Right tragion-pronasale (t-Prn[R])	Distance between tragion and pronasale (right side)
7	Left tragion- pronasale (t-Prn[L])	Distance between tragion and pronasale (left side)
8	Right tragion-pogonion (t-Pog[R])	Distance between tragion and pogonion (right side)
9	Left tragion-pogonion (t-Pog[L])	Distance between tragion and pogonion (left side)
10	Right gonion-pogonion (Go-Pog[R])	Distance between gonion and pogonion (right side)
11	Left gonion-pogonion (Go-Pog[L])	Distance between gonion and pogonion (left side)
12	Bizygomatic width (BZW)	Distance between the right and left zygion
13	Cheilion[R]- Cheilion [L] {Ch(R)-Ch(L)}	Distance between the right and left cheilion

Inter-landmark distances (Fig. 2) were measured for each participant using digital calipers (Marita Digital Caliper; Martay, India) and recorded manually.

**Figure 2:**
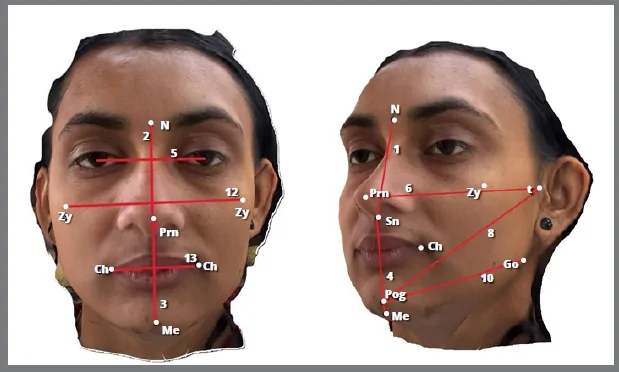
Distances measured. 1) Nasion-subnasale (N-Sn); 2) Nasion-pronasale (N-Prn); 3) Pronasale-menton (Prn-Me); 4) Subnasale-menton (Sn-Me); 5) Interpupillary distance (ITP); 6) Tragion-Pronasale (t-Prn); 8) Tragion-Pogonion (t-Pog); 10) Gonion-pogonion (Go-Pog).

For the LiDAR-based mobile application scanning method, the iPhone 15 Pro with the Polycam application (iOS version 5.0.1; Polycam Inc., San Francisco, CA, USA) was employed. This application generates a mesh to construct a three-dimensional (3D) model from photographs captured within the app. During scanning, the operator moved 180° around the participant capturing images. The Polycam application processed these images to create a 3D model of the participant’s face. The application includes a built-in function for measuring distance between points. After calibration, linear measurements were recorded (Fig. 3A).

**Figure 3:**
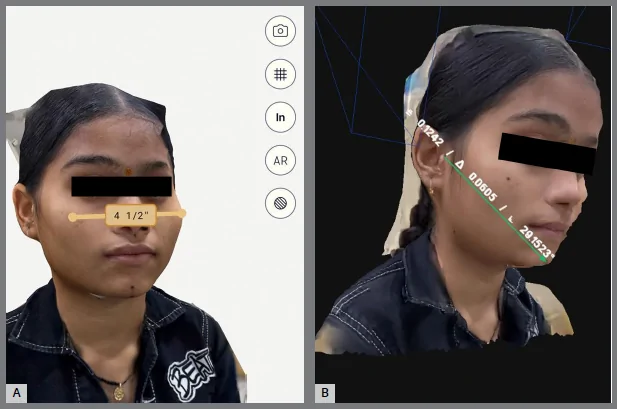
A) 3D facial scans created with LiDAR-based Polycam app. B) 3D facial scans created with SCP based 3Df Zephyr application.

Using identical facial markings, the operator recorded a video of the patient’s face while moving 180 degrees around them. The recorded video was subsequently imported into 3D Zephyr software (3DFLOW, Udine, Italy), which processed the footage to generate a facial mesh and construct a (3D model of the patient’s face. Following the calibration, the distances between the marked facial points were systematically recorded (Fig. 3B). The soft tissue measurements obtained using the three methods were tabulated, and the data were subjected to statistical analysis.

## RESULTS

Data were tabulated in MS Excel and analyzed using SPSS v23 software with significance level at 5%. Data underwent normality testing using Kolmogorov-Smirnov test. Parametric tests were applied for normally distributed data. Intraexaminer reliability was assessed using intraclass correlation coefficient test. Comparisons among groups were performed using one-way ANOVA with Tukey *post-hoc* test. In examining intra-examiner reliability across manual, LiDAR, and SCP methods, manual approach showed high consistency (0.82-0.99), LiDAR demonstrated good reliability (0.77-0.99), while SCP ranged from 0.61 to 0.99, showing lowest reliability for Ch(R)-Ch(L).

Table 3 compares measurements using these three methods. Results indicated no statistically significant differences for most parameters except for cheilion measurement [Ch(R)-Ch(L)], in which significant differences were found between LiDAR and SCP methods.

**Table 3:** Comparison of measurements between three methods.

Variable	Manual	LiDAR	SCP	p- value
	Mean±SD (in mm)	Mean±SD (in mm)	Mean±SD (in mm)	
N-Sn	59.92±5.33	58.88±5.60	59.99±5.89	0.643
N-Prn	52.71±3.56	52.55±2.86	54.21± 3.79	0.082
Prn-Me	68.02±6.90	68.07±6.73	69.05± 8.11	0.794
Sn-Me	57.11±4.22	57.29±3.56	58.5± 4.70	0.306
ITP	64.38±4.82	63.46±4.57	65.91± 6.43	0.150
t-Prn (R)	126.35±9.96	128.28±9.86	125.61± 10.68	0.520
t-Prn (L)	125.77±9.01	125.43±9.24	127.89± 9.64	0.481
t-Pog (R)	129.78±9.94	131.76±8.91	129.35± 11.41	0.563
t-Pog (L)	131.03±8.83	131.32±8.55	132.01± 9.69	0.893
Go-Pog (R)	93.07±12.27	93.95±12.46	97.26± 10.42	0.285
Go-Pog (L)	93.72±12.82	94.99±11.81	99.08± 10.80	0.139
BZW	104.92±10.11	104.96±11.95	106.79± 8.70	0.683
Ch(R)-Ch(L)	49.55 ±3.46^a,b^	48.88±2.94^a^	50.93± 3.51^b^	0.031*

* Significant difference at p≤0.05. Letters indicate pairwise comparison: same letter in a row indicates non-significant difference between two groups, different letter in a row indicates significant difference between the two groups.

Figure 4 analyzes absolute error of LiDAR and SCP measurements, compared to manual technique. LiDAR errors ranged from -0.04 to -1.98, while SCP ranged from -0.07 to -5.36. LiDAR showed least error for Pronasale-menton (Prn-Me), while SCP showed least error for Nasion-subnasale (N-Sn). Maximum errors were in right tragion-pogonion (t-Pog (R)) for LiDAR and left gonion-pogonion (Go-Pog (L)) for SCP. For certain measurements (N-Sn, t-Prn (R), and t-Pog (R)), LiDAR showed greater error than SCP, while other measurements showed higher error in SCP methodology.

**Figure 4:**
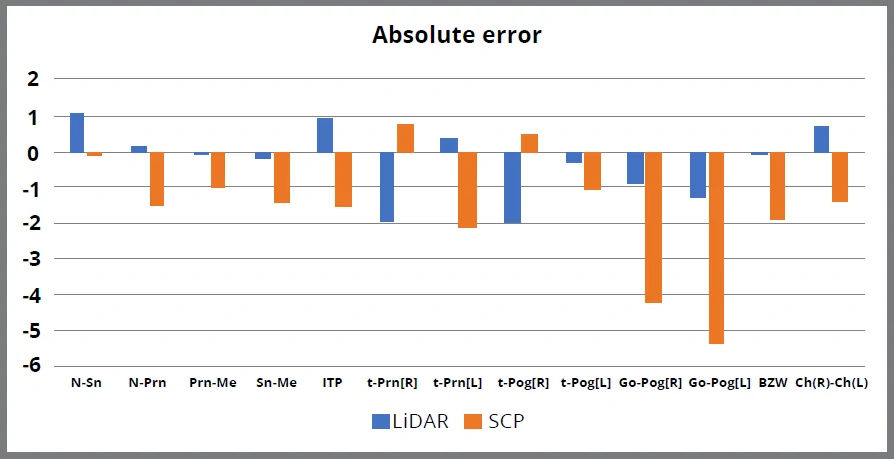
Absolute error.

Figure 5 presents the absolute percentage error for measurements using LiDAR and SCP, compared to the manual method. The absolute percentage error for LiDAR ranged from 0.02 to -1.68, while for SCP, it ranged from -0.10 to -6.31. Negative values indicate underestimation, while positive values show overestimation. The maximum absolute percentage error for LiDAR was -1.68 for Go-Pog(L), whereas SCP showed -6.31 for the same variable. The minimum error for LiDAR was 0.02 for BZW, compared to -0.10 for N-Sn in SCP. LiDAR generally showed lower mean absolute percentage errors than SCP, indicating higher accuracy for most facial dimensions. However, for variables like N-Sn, t-Prn (R), and t-Pog (R), SCP demonstrated better accuracy than LiDAR.

**Figure 5:**
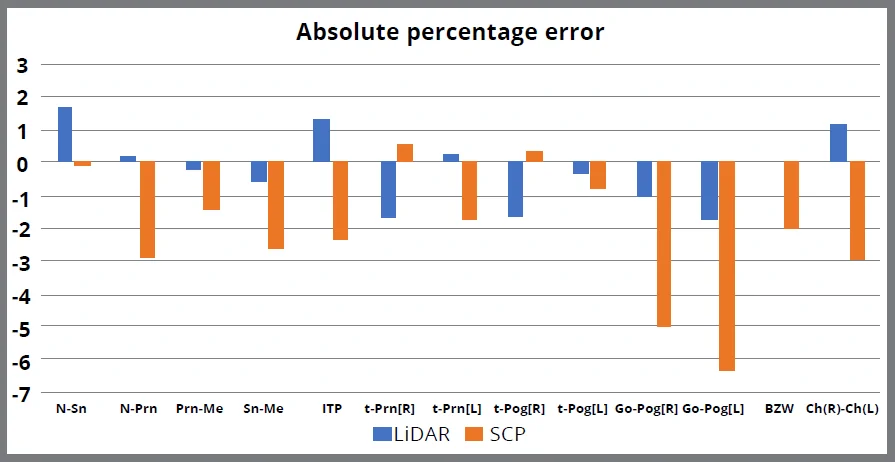
Absolute percentage error.

## DISCUSSION

The primary aim of this study was to evaluate and compare two distinct methodologies for capturing three-dimensional facial scans: single-camera photogrammetry (SCP) and a mobile application utilizing LiDAR-based three-dimensional facial scanning technology. To determine the precision of the identified soft tissue, measurements obtained through manual techniques were employed as benchmarks for comparative evaluation. Previous studies have utilized cadavers^7^ or mannequins^8^ ^,^ ^9^ to compare various scanning methodologies, in order to assess the reproducibility of soft tissue landmarks. While stationary objects, cadaver heads, and plastic models can be beneficial for certain types of research, they do not fully encapsulate the complexities of human faces. Human faces are dynamic and change with expressions, making accurate scanning challenging. The study involved 36 healthy patients to assess three-dimensional facial scanning efficacy. Errors can arise when comparing indirect measurements from images to direct physical measurements. According to Bianchi et al.^10^, the primary reason for these errors is the challenge of accurately marking and identifying anatomical points on the images. Therefore, in this study, a direct measurement method was selected to mitigate errors. In the present study, when intraexaminer reliability was assessed, the manual approach demonstrated high consistency and exhibited the least variability. While LiDAR technology was generally reliable, it did not perform as well as the manual method. SCP was the least consistent. The means of most measurements were comparable across the three methods, with non-significant differences; however, there was one notable exception in the results: the measurement of the distance between the cheilions (R&L). Significant differences were observed in this variable between the LiDAR and SCP methods. This finding aligns with a previous study by Seifert et al.^6^, which suggested that, due to the mouth being a flexible area, it was more challenging for smartphone scanning applications to capture it accurately, resulting in larger deviations.

In this study, the mean absolute error and the mean absolute percentage error were assessed. The SCP method exhibited the most pronounced errors for both the right and left gonion-pogonion variables (Go-Pog (L) and (R)). These results were consistent with those reported by Staller et al.^4^, wherein the distances from the right and left gonions to the pogonion demonstrated clinically significant disparities between single-camera photogrammetry (SCP) and multi-camera photogrammetry (MCP) methods, as well as between SCP and Direct anthropometry (DA). The SCP method demonstrated the highest levels of error, which may be ascribed to either the misidentification of anatomical landmarks or magnification of this specific region. There were several instances in which shadowing effects in this area caused the markings to appear either too small or obscured by freckles on participants’ skin. It has been noted that the SCP method exhibits reduced accuracy when measuring regions characterized by greater curvature, particularly with respect to the gonial angle. Similar findings were reported by Hong et al.^11^, who determined that regions with curvature exhibited the most substantial errors. The SCP method tends to underestimate measurements to a greater extent than LiDAR, indicating greater measurement error in the SCP for the lower face region and mandibular length (Go-Pog).

The significant errors of LiDAR and SCP in measuring the tragion-pronasale [t-Prn (R)] and tragion-pogonion [t-Pog (R)] stem from various technical, anatomical, and methodological factors. As the left and right sides of the face are not perfectly symmetrical, minor errors in landmark detection can lead to inconsistencies. Additionally, the LiDAR and SCP process depths differ, which may result in greater variations in specific landmarks.

LiDAR employs structured light or laser pulses, offering higher depth precision, but is susceptible to surface reflectivity issues. In contrast, SCP reconstructs 3D images from 2D photographs, making it more vulnerable to perspective distortions, particularly in lateral landmarks, such as t-Prn (R) and t-Pog (R).

The mean absolute error and mean absolute percentage error associated with ITP were significant for both scanning techniques used in this study. However, it was observed that the SCP method exhibited a comparatively greater error (-2.30 ± 4.62). This observation is consistent with the findings of previous research by Staller et al.^4^, which attributed these discrepancies to the difficulties in precisely identifying the center of the pupil during each measurement.

The variability distinguished in subnasale measurements was similarly emphasized in studies conducted by Staller et al.^4^ and Chong et al.^12^, wherein the results revealed an expanded margin of error. This variability may be attributed to various anatomical and methodological factors. A salient aspect is the difficulty associated with accurate identification of the subnasale. Furthermore, fluctuations in lip posture, in conjunction with the morphology of both the nose and lips, exacerbate this challenge, as these anatomical features can obscure the subnasale landmark. This clarifies the rationale behind the augmented error in measurements pertaining to the Sn variable, as evidenced by N-Sn (1.04±2.83 mm) for the LiDAR method and Sn-Me (-1.40± 3.12 mm) for the SCP method.

The challenges associated with measurements involving the nasion can likely be ascribed to the distinctive positioning of the nasion at the intersection of multiple facial planes, rendering it a challenging target for smartphone scanning technologies, as concluded in the investigation conducted by Seifert et al.^6^ Gwilliam et al.^13^ assessed the reproducibility of soft tissue landmarks using 3D facial scans made using laser scanners, and reported increased errors in the reproducibility of soft tissue nasion. As a result, the noticeable increase in absolute errors for N-Sn measurements (1.04±2.83 mm) using LiDAR and N-Prn measurements (-1.50±2.61 mm) with the SCP method can be reasonably explained.

SCP often struggles to accurately capture side-profile landmarks, due to variations in the camera angles and lighting conditions. If the camera does not capture the profile precisely, the software may miscalculate the 3D landmark position, leading to large discrepancies. Additionally, depth perception of the SCP can be affected by shadows or occlusions, particularly in areas such as t-Pog (L), where visibility varies depending on lighting.

Although LiDAR is generally more accurate for in-depth capture, it can still encounter difficulties with curved surfaces, causing some measurement deviations. Furthermore, different software algorithms process facial scans differently, implying that LiDAR and SCP may not be able to identify landmarks at the same position, resulting in inconsistencies. Even slight variations in landmark placement can lead to noticeable deviations in final measurements.

In the present investigation, it was determined that, for the majority of the facial metrics examined, the LiDAR methodology exhibited a lower mean absolute percentage error than the SCP methodology. This indicates that, upon comparing the measurements acquired through both methodologies with the actual measurements, LiDAR demonstrated superior accuracy for particular facial dimensions. This suggests that LiDAR technology, as employed in the Polycam mobile application, may represent a more efficacious option for precise measurement of facial dimensions than SCP.

Numerous prior investigations have compared mobile scanning applications utilizing TrueDepth sensors and standard commercially accessible three-dimensional facial scanners. Pellitteri et al.^5^ assessed the accuracy of digital scans obtained using the Structured Light Scanner-based Face Hunter facial scanner and the Dental Pro application, which utilizes TrueDepth camera technology integrated into Apple devices. Their findings revealed that both scanning modalities can be effectively utilized in clinical environments, leading to the conclusion that mobile scanning applications are reliable, which aligns with the outcomes of the current study.

D’Ettorre et al.^3^ conducted a comparative analysis of the stereophotogrammetry 3dMD system and TrueDepth technology, with a particular focus on the Bellus3D Face Application and Capture application. The study concluded that the 3dMD system was characterized by its rapidity and precision; however, it was also regarded as being bulky and expensive, necessitating a higher degree of operator expertise and patient compliance due to protracted acquisition times. In addition, the authors concluded that smartphone applications provide enhanced portability and cost-effectiveness, and also substantiate the reliability of mobile-based applications, leading to conclusions that correspond with those of the present study.

Andrews et al.^14^, investigated the efficacy of smartphone-based photogrammetry, specifically the Bellus3D Face application, for the acquisition of three-dimensional facial images in comparison to the stereophotogrammetry technique, 3dMD, and similarly demonstrated findings in concordance with the present study’s conclusions.

In their systematic review, Quinzi et al.^15^ examined diverse methodologies employed in various studies to compare different technologies pertinent to facial digitization. They concluded that although mobile-based devices performed less effectively on irregular and deep surfaces, all devices were deemed sufficiently accurate for clinical applications. Internal or external depth-sensing cameras with smartphones enhanced accuracy but required more operator skill and longer acquisition times. Stationary stereophotogrammetry remained the gold standard for accuracy and efficiency, minimizing artifacts. However, their findings differed from the current study. The accuracy variations stem from using an older iPhone in previous studies, as technology evolves rapidly. The older iPhone lacked LiDAR technology, which captures more detailed facial surface information for precise scans. Without this technology, older iPhones could not capture details effectively, resulting in less accurate scans.

At the time this research was conducted, only one study by Seifert et al.^6^ has assessed the accuracy of three smartphone-based applications (ScandyPro, EM3D, and Polycam) compared to a stationary 3DMD system, which is regarded as the gold standard in facial scanning. The smartphone apps used Apple’s LiDAR technology on an iPhone 14 Pro to capture facial data. Accuracy was assessed by comparing the scanned surfaces to a reference model, using metrics such as the maximal distance (MaxD) and mean surface distance (MSD) for each landmark. In contrast, our study focused on evaluating a single LiDAR-based app, Polycam, and compared it with SCP using manual measurements as the gold standard. Accuracy and reliability were measured using the mean absolute error (AE), mean absolute percentage error (APE), and intraclass correlation coefficient (ICC). Both studies found that smartphone-based 3D scanning apps offer a practical and cost-effective way to capture detailed facial images, which could enhance patient care, particularly in settings where advanced equipment is not accessible. Overall, this technology represents a significant improvement in the manner in which facial images are obtained for medical purposes.

## CONCLUSIONS

One significant benefit of LiDAR-based smartphone scanning is eliminating complex machinery or extensive training needs. While photogrammetry software (3DF Zephyr) in SCP methodology requires specialized training, smartphone applications are designed to be intuitive. Individuals with minimal smartphone experience can quickly learn these applications, similar to how people easily learn phone photography. Such scanning systems are particularly beneficial in resource-limited areas.

LiDAR technology is more reliable for 3D facial scanning but has limitations in insufficient light. The SCP is less accurate, especially for lower face landmarks, and needs careful calibration. While both methods provide comparable results, technology selection should depend on specific requirements.

## Data Availability

Data restricted for ethical or legal reasons: Due to the sensitive nature of the data and ethical restrictions, the datasets supporting this study are not publicly available.

## References

[1] Mai HN, Lee DH (2020). Accuracy of mobile device-compatible 3D scanners for facial digitization Systematic review and meta-analysis. J Med Internet Res.

[2] Piedra-Cascón W, Meyer MJ, Methani MM, Revilla-León M (2020). Accuracy (trueness and precision) of a dual-structured light facial scanner and interexaminer reliability. J Prosthet Dent.

[3] D'Ettorre G, Farronato M, Candida E, Quinzi V, Grippaudo C (2022). A comparison between stereophotogrammetry and smartphone structured light technology for three-dimensional face scanning. Angle Orthod.

[4] Staller S, Anigbo J, Stewart K, Dutra V, Turkkahraman H (2022). Precision and accuracy assessment of single and multicamera three-dimensional photogrammetry compared with direct anthropometry. Angle Orthod.

[5] Pellitteri F, Brucculeri L, Spedicato GA, Siciliani G, Lombardo L (2021). Comparison of the accuracy of digital face scans obtained by two different scanners. Angle Orthod.

[6] Seifert LB, Beyer M, Czok V, Aigner A, Abazi S, Thieringer FM (2024). Comparative accuracy of stationary and smartphone-based photogrammetry in oral and maxillofacial surgery a clinical study. J Clin Med.

[7] Fourie Z, Damstra J, Gerrits PO, Ren Y (2011). Evaluation of anthropometric accuracy and reliability using different three-dimensional scanning systems. Forensic Sci Int.

[8] Wellens HLL, Hoskens H, Claes P, Kuijpers-Jagtman AM, Ortega-Castrillón A (2020). Three-dimensional facial capture using a custom-built photogrammetry setup Design, performance, and cost. Am J Orthod Dentofacial Orthop.

[9] Kühlman DC, Almuzian M, Coppini C, Alzoubi EE (2023). Accuracy (trueness and precision) of four tablet-based applications for three-dimensional facial scanning An in-vitro study. J Dent.

[10] Bianchi SD, Spada MC, Bianchi L, Verzè L, Vezzetti E, Tornincasa S (2004). Evaluation of scanning parameters for a surface colour laser scanner. Int Congr Ser.

[11] Hong C, Choi K, Kachroo Y, Kwon T, Nguyen A, McComb R (2017). Evaluation of the 3dMDface system as a tool for soft tissue analysis. Orthod Craniofac Res.

[12] Chong Y, Liu X, Shi M, Huang J, Yu N, Long X (2021). Three-dimensional facial scanner in the hands of patients validation of a novel application on iPad/iPhone for three-dimensional imaging. Ann Transl Med.

[13] Gwilliam JR, Cunningham SJ, Hutton T (2006). Reproducibility of soft tissue landmarks on three-dimensional facial scans. Eur J Orthod.

[14] Andrews J, Alwafi A, Bichu YM, Pliska BT, Mostafa N, Zou B (2023). Validation of three-dimensional facial imaging captured with smartphone-based photogrammetry application in comparison to stereophotogrammetry system. Heliyon.

[15] Quinzi V, Polizzi A, Ronsivalle V, Santonocito S, Conforte C, Manenti RJ (2022). Facial scanning accuracy with stereophotogrammetry and smartphone technology in children a systematic review. Children (Basel).

